# Vaginal Mucormycosis: A Case Report

**DOI:** 10.1155/S1064744901000205

**Published:** 2001

**Authors:** Jack D. Sobel

**Affiliations:** 1 Division of Infectious Diseases Detroit Medical Center Wayne State University School of Medicine Detroit MI USA; 2 Division of Infectious Diseases 4 Brush Harper University Hospital 3990 John R Detroit MI 48201 USA

## Abstract

Although Zygomycetes cause life-threatening, opportunistic infections in immunocompromised hosts, the first case of vaginitis caused by *Mucor* species in a healthy woman is reported. *Mucor* vaginitis, which caused mild symptoms only, was refractory to conventional azole therapy and resistant to flucytosine. Cure was achieved with topical amphotericin B.


Infect Dis Obstet Gynecol 2001;9:117–118

### Vaginal mucormycosis: a case report

**Jack D. Sobel**

*Division of Infectious Diseases, Detroit Medical Center, Wayne State University School of Medicine, Detroit, MI*

Although Zygomycetes cause life-threatening, opportunistic infections in immunocompromised hosts, the first case of vaginitis caused by *Mucor* species in a healthy woman is reported. *Mucor* vaginitis, which caused mild symptoms only, was refractory to conventional azole therapy and resistant to flucytosine. Cure was achieved with topical amphotericin B.

Key words: VAGINITIS, FUNGAL INFECTION, AMPHOTERICIN B

The vast majority of vaginal fungal infections are caused by *Candida* species, especially *Candida albicans*[1,2]. Recently, attention has focused on an unconfirmed increase in vaginal infections caused by *C. glabrata*[3]. Most microbiological investigations of large numbers of women with fungal vaginitis have reported infrequent symptomatic vaginitis caused by unusual species of *Candida* including *C. tropicalis, C. parapsilosis*, and *C. krusei*[1,3]. Rarely, vaginitis caused by *Saccharomyces cerevisiae* has been reported[4]. Although the Zygomycetes class of microorganisms are recognized as major pathogens in immunocompromised hosts, no mention in the literature has been made of vaginitis caused by these opportunistic pathogens[5].

### CASE HISTORY

A 56-year-old female was referred because of vulvovaginal symptoms that had begun approximately 18 months previously, consisting predominantly of a stinging sensation in the vulva. Other symptoms included vulvovaginal itching, soreness, and discomfort. Because of chronic symptoms and positive 10% KOH microscopy, she had received repeated courses of topical (miconazole, clotrimazole and terconazole) and systemic azoles (fluconazole) as well as topical boric acid without any relief. On one occasion, a fungal culture obtained was positive for *Mucor* species. She had no abnormal discharge or dyspareunia and her husband was asymptomatic. Prior to the onset of symptoms, she had repeated attacks of bacterial cystitis, treated with antibiotics. Her past medical history was significant only for interstitial cystitis.

Examination demonstrated normal appearance of vulva and vestibule. The vagina contained fairly copious, pasty white discharge, which was not malodorous and was otherwise normal; the cervix was normal. Laboratory tests showed a vaginal pH of 4.2 with a negative amine test. Saline microscopy revealed large numbers of atypical blastocondia and broad, non-septate hyphae branching at irregular intervals. A 10% KOH examination revealed large numbers of blastocondia and hyphal elements consistent with Zygomycetes. Definitive diagnosis was established by culture, which showed *Mucor indicus* species. *In vitro* susceptibility tests showed that the vaginal isolate was sensitive to amphotericin B and resistant to flucytosine, imidazoles and triazoles. She was placed on topical therapy with 3% amphotericin B cream 5 g daily

Correspondence to: J. D. Sobel, MD, Division of Infectious Diseases, 4 Brush, Harper University Hospital, 3990 John R, Detroit, MI 48201. E-mail: Jsobel@intmed.wayne.edu for 3 weeks. After an initial clinical response to amphotericin B, she had a relapse with minimal symptoms and cultures remained positive. She was retreated with 4 additional weeks of topical amphotericin B and then placed on a tapering alternate day dose for 2 months. Her symptoms of stinging and itching resolved and she has remained culture-negative and asymptomatic for a further 12-month follow-up period.

### COMMENTS

Mucormycosis is a rare but commonly fatal invasive fungal infection caused by one of the ubiquitous saprophytic fungi of the Zygomycetes class. Species of the *Rhizopus, Mucor*, and *Absidia* genera of the Mucoraceae family are the organisms most commonly isolated[5,6]. Mucormycosis is known to affect patients who are immunocompromised and usually neutropenic, and those with uncontrolled diabetes mellitus and ketoacidosis[6]. Other risk factors include iron overload, desferrioxamine administration, wound contamination with soil, colonized adhesive tape, corticosteroid use and severe protein calorie malnutrition. Clinical features depend upon the organ involved and most commonly involve paranasal sinuses and lungs and less commonly the gastrointestinal tract[7]. There are few reports of genitourinary mucormycosis in the literature. Genitourinary involvement is predominantly of the kidneys and has been found with systemic dissemination[8]. Parenteral high-dose amphotericin B is the most effective treatment. No case of vaginal infection by Zygomycetes has been published to date.

The lack of response in the reported patient to treatment with topical azoles is not surprising, since these agents are inactive against *M. indicus* and the Mucoraceae in general. Similarly, flucytosine, which is useful in eliminating azole-resistant *C. glabrata*, has no activity *in vitro* against Zygomycetes. Amphotericin B remains the mainstay of therapy in life-threatening mucormycosis, but has been used extremely infrequently as a topical agent for vaginal mycotic infections and is not available commercially for this indication. A 3% concentration was used somewhat arbitrarily, since this concentration is used to treat oropharyngeal candidiasis due to sensitive *Candida* species. Response in the reported case was not dramatic and required retreatment and extension of the treatment duration. The pathogenesis of this obscure infection remains an enigma and could not be correlated with any personal habits (douching) or other risk factors. This unusual and obstinate case emphasizes the value of obtaining vaginal fungal cultures in treatment of unresponsive cases, as well as the need for full identification of the isolated pathogen and basing therapy on susceptibility testing.

### REFERENCES

1. Odds FC. Candidosis of the genitalia. In Odds FC. *Candida and Candidiasis. A Review and Bibliography*, 2nd edn. Philadelphia: Baillière Tindall, 1998:124–36
2. Sobel JD. Epidemiology and pathogenesis of recurrent vulvovaginal candidiasis. *Am J Obstet Gynecol* 1985;152:924–34
3. Horowitz BJ, Giaquinta D, Ito S. Evolving pathogens in vulvovaginal candidiasis. Implications for patient care. *J Clin Pharmacol* 1992;32:248–55
4. Sobel JD, Vazquez J, Lynch M, *et al.* Vaginitis due to *Saccharomyces cerevisiae*. Epidemiology, clinical aspects and therapy. *Clin Infect Dis* 1993;16:93–9
5. Sugar AM. Mucormycosis. *Clin Infect Dis* 1992;14:(Suppl):126-9
6. Sugar AM. Agents of mucormycosis and related species. In Mandell GL, Bennett JE, Dolin R, eds. *Principle and Practice of Infectious Diseases*, 4th edn. New York: Churchill Livingston,1995:2311–21
7. Rangel-Guerra RA, Martinez HR, Saenz C, *et al.* Rhinocerebral and systemic mucormycosis: clinical experience with 36 cases. *J Neurosci* 1996;143:19–30
8. Pastor Poru E, Martinezleon MI, Alvarez Bustos G, *et al.* Isolated renal mucormycosis in two patients with AIDS: case report. *Am J Roentgenol* 1996;166:1282–6

Received 01/04/01; Accepted 01/17/01
